# Protection against Enterovirus 71 with Neutralizing Epitope Incorporation within Adenovirus Type 3 Hexon

**DOI:** 10.1371/journal.pone.0041381

**Published:** 2012-07-27

**Authors:** Xingui Tian, Xiaobo Su, Xiao Li, Haitao Li, Ting Li, Zhichao Zhou, Tianhua Zhong, Rong Zhou

**Affiliations:** 1 State Key Lab of Respiratory Disease, The First Affiliated Hospital of Guangzhou Medical College, Guangzhou, Guangdong, China; 2 Department of Medical Genetics and Cell Biology, School of Basic Science, Guangzhou Medical College, Guangzhou, Guangdong, China; Aaron Diamond AIDS Research Center with the Rockefeller University, United States of America

## Abstract

Enterovirus 71 (EV71) is responsible for hand, foot and mouth disease with high mortality among children. Various neutralizing B cell epitopes of EV71 have been identified as potential vaccine candidates. Capsid-incorporation of antigens into adenovirus (Ad) has been developed for a novel vaccine approach. We constructed Ad3-based EV71 vaccine vectors by incorporating a neutralizing epitope SP70 containing 15 amino acids derived from capsid protein VP1 of EV71 within the different surface-exposed domains of the capsid protein hexon of Ad3EGFP, a recombinant adenovirus type 3 (Ad3) expressing enhanced green fluorescence protein. Thermostability and growth kinetic assays suggested that the SP70 epitope incorporation into hypervariable region (HVR1, HVR2, or HVR7) of the hexon did not affect Ad fitness. The SP70 epitopes were thought to be exposed on all hexon-modified intact virion surfaces. Repeated administration of BALB/c mice with the modified Ads resulted in boosting of the anti-SP70 humoral immune response. Importantly, the modified Ads immunization of mother mice conferred protection in vivo to neonatal mice against the lethal EV71 challenge, and the modified Ads-immunized mice serum also conferred passive protection against the lethal challenge in newborn mice. Compared with the recombinant GST-fused SP70 protein immunization, immunization with the Ads containing SP70 in HVR1 or HVR2 elicited higher SP70-specific IgG titers, higher neutralization titers, and conferred more effective protection to neonatal mice. Thus, this study provides valuable information for hexon-modified Ad3 vector development as a promising EV71 vaccine candidate and as an epitope-delivering vehicle for other pathogens.

## Introduction

Enterovirus 71 (EV71) is the most frequently detected pathogen in hand, foot and mouth disease (HFMD) patients complicated with the severest forms of neurological disorders [1], [2], [3]. Outbreaks of EV71 have been reported around the world since 1969. Especially since the late 1990s, there has been a significant increase in EV71 epidemics, and it has emerged as a serious threat to public health throughout the Asia-Pacific region [4], [5], [6], [7]. However, there are no effective antiviral drugs and vaccines presently available. The development of effective vaccines is a top priority in terms of control strategies [8]. EV71 is a small, non-enveloped, positive single-stranded RNA virus with four capsid proteins: VP1, VP2, VP3 and VP4. The neutralizing antibodies elicited by SP70 epitope containing amino acids 208–222 of VP1 protein were able to confer good *in vivo* passive protection against homologous and heterologous EV71 strains in suckling Balb/c mice [9], [10], [11]. Therefore, the epitope-based vaccine represents a promising candidate for EV71.

Epitope-based vaccination is one area under intense investigation for the delivery of precise vaccine components to the immune system. The peptide epitope represents the minimal immunogenic region of a protein antigen and allows exquisite direction and control of immune responses [12]. However, there are some drawbacks including poor immunogenicity of the simple peptide and the need to potently stimulate T cells and elicit immunological memory. Although some approaches, such as adjuvant science, lipopeptide conjugation, direct delivery to dendritic cells, and particulate delivery systems have been developed, novel and powerful methods for efficiently delivering epitopes are still needed [12], [13], [14].

Adenovirus (Ad), especially Ad serotype 5 (Ad5) vectors, have been successfully used for a variety of vaccine applications, including cancer and infectious diseases [15], [16], [17], [18]. Recently a novel approach is developed to incorporate antigenic epitopes into the Ad capsid proteins: hexon, fiber knob, and penton base, as well as protein IX [19], [20], [21], [22]. Incorporating immunogenic peptides into the Ad capsid offers potential advantages: a strong humoral response similar to the response generated by native Ad capsid proteins, allowing boosting of the immune response against antigenic epitopes that are part of the Ad capsid [18]. Hexon is the largest and most abundant capsid protein. Although several groups have shown that short heterologous peptides can be incorporated into the Ad5 hexon without affecting the virion’s stability or function [19], [22], [23], [24], [25], hexon modification often results in failure of rescuing viruses or poorly growing viruses, suggesting hexon modification may interfere with viral formation [24], [26], [27]. The immune response against an epitope inserted into Ad5 hexon is dependent on the incorporation site and sometimes not satisfying [28]. So it is necessary to develop non-Ad5 vector as an epitope-delivering system.

Here we reported a noval epitope-delivering system based Ad3. An Ad3 vector, a member of species B adenoviruses, has been developed previously as a candidate for vaccine design and gene transfer [29]. Ad3-based vectors are relatively safe as compared to Ad5 [30]. Unlike members of other adenovirus species that bind to the cell surface receptor CAR, members of species B recognize the membrane co-factor proteins CD46, CD80, and CD86 as cellular receptors [31], [32]. In this study, we invested whether foreign peptides could be incorporated into different surface-exposed domains of the Ad3 hexon without affecting the normal function and whether they could elicit effective immune responses. We developed an epitope-based vaccine against EV71 by incorporating the foreign epitope EV71-derived SP70 containing 15 amino acids within the Ad3 hexon. Our study provides valuable information for the development of Ad3-based vaccine delivering epitopes from pathogens or cancer cells by hexon-incorporation.

## Results

### Construction of Hexon-modified Ad3 Containing EV71 Epitope

**Figure 1:**
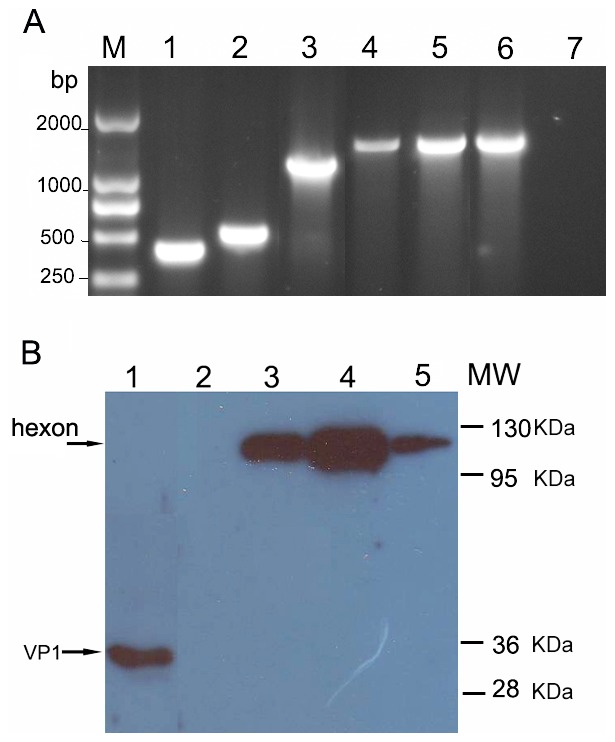
SP70 epitope was incorporated in hexon hypervariable regions 1, 2, or 7. (A) Rescued viruses were amplified, and viral DNA was analyzed to confirm stable modification of hexon genes. Hexon-specific PCR primers HexU and SP70r, Hu1 and Hr1 confirmed incorporation of coding regions for SP70 epitope inserts at the hexon HVR1, HVR2, and HVR7 sites. Lane 1, 2, and 3 were R1SP70A3, R2SP70A3, and R7SP70A3, respectively, with primers HexU and SP70r. Lane 4, 5, and 6 were R1SP70A3, R2SP70A3, and R7SP70A3, respectively, with primers Hu1 and Hr1. Lane 7, negative control Ad3EGFP with primers HexU and SP70r. (B) Purified EV71-08-02 strain (Lane 1), R1SP70A3 (Lane 3), R2SP70A3 (Lane 4), R7SP70A3 (Lane 5), or unmodified Ad3EGFP (Lane 2) were separated on SDS-PAGE gel. The proteins were transferred to polyvinylidene fluoride (PVDF) membrane, and then stained with anti-SP70-GST antibody. The arrow indicates SP70 protein molecularly incorporated into the hexon protein.

The hexon-modified plasmids, pR1SP70A3egf, pR2SP70A3egf, pR4SP70A3egf, pR5SP70A3egf, pR7SP70A3egf were obtained by homologous recombination and confirmed by restriction enzyme digestions and full length hexon sequencing analysis (data not shown). In the case of R1SP70A3, R2SP70A3, and R7SP70A3, the replacement of epitopes resulted in viable viruses. The fluorescence focus did spread from individual cells transfected with the pR4SP70A3egf, however, R4SP70A3 grew much more slowly than did the other viruses, and repeated attempts to amplify and purify the virion by CsCl centrifugation were unsuccessful. In cells transfected with the pR5SP70A3egf, no evidence of virus growth was seen even at 14 days post-transfection. So the viruses of R1SP70A3, R2SP70A3, and R7SP70A3 were chosen for further characterization. The hexon modification of viruses of R1SP70A3, R2SP70A3, and R7SP70A3 were confirmed by PCR and sequencing using genomic DNA from the purified virions (Fig. 1A). In order to determine if the hexon-modified vectors were presenting the SP70 epitope within the hexon region, purified R1SP70A3, R2SP70A3, R7SP70A3, or unmodified Ad3EGFP were subjected to Western blot analysis with anti-SP70-GST antibody. The SP70 peptide was detected as an approximately 120 kDa protein band associated with R1SP70A3, R2SP70A3, R7SP70A3 particles (Fig. 1B, lanes 3, 4 and 5, respectively). The size of the 120 kDa band corresponded to the expected size of the Ad3 hexon protein with SP70 peptide genetically incorporated. There was no band detected on Ad3EGFP particles (Fig. 1B, lane 2), HEp-2 cells or Vero cells as negative controls (data not shown). The size of band detected in purified EV71-08-02 strain corresponded to the expected size 32.6 kDa of the EV71 VP1 peptide (Fig. 1B, Lane 1).

**Figure 2:**
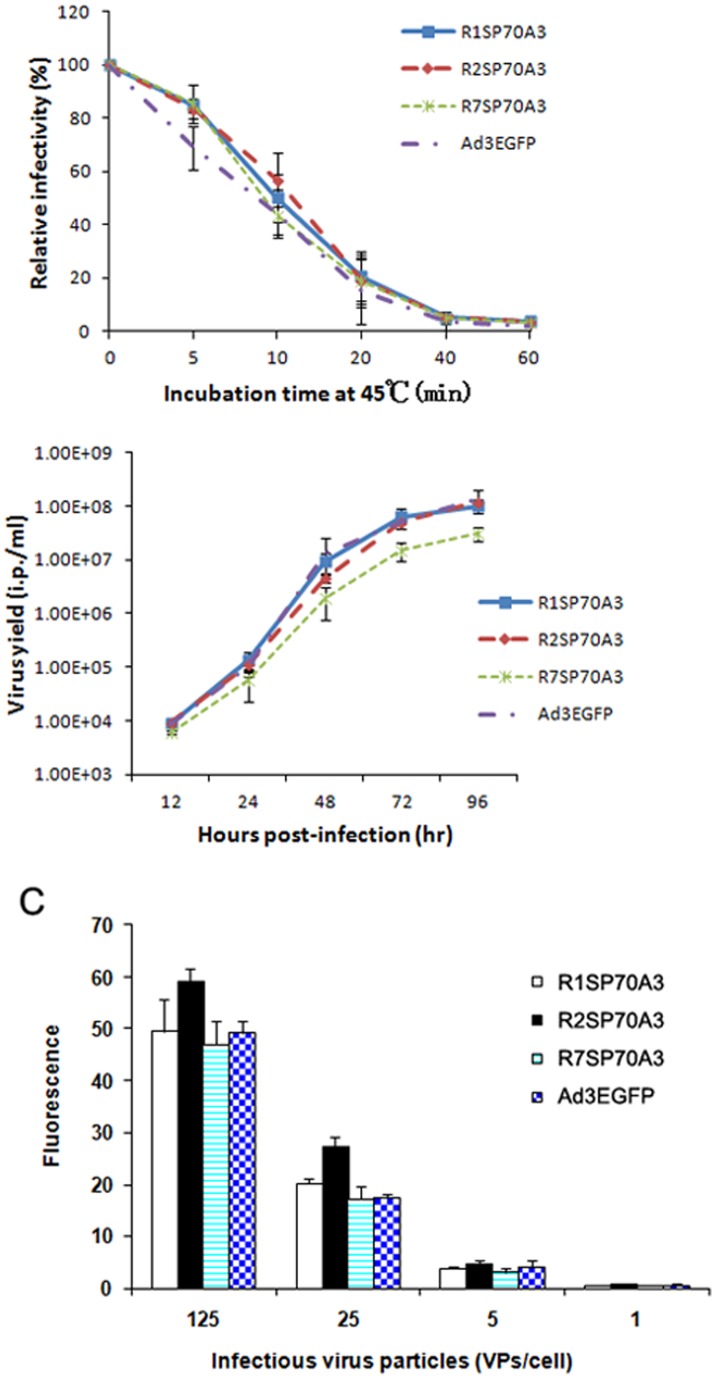
Characteristics of hexon-modified Ad vectors. (A) Thermostability assay of modified Ad vectors. Viruses were incubated at 45°C for 0, 5, 10, 20, or 40 min. The number of infectious particles remaining in each sample was determined by fluorescent focus assay. Viability was calculated as infectious particles remaining in each sample as a percent of the number of infectious particles in samples at the zero time point. (B) Growth kinetics of modified Ad vectors. Cells were infected with the viruses respectively at 5 VPs/cell and harvested with medium at different time points. The number of infectious particles at each time point was determined by fluorescent focus assay. (C) Gene transfer efficacies of modified Ad vectors. Fluorescence of HEp-2 cells infected with the viruses respectively at 1, 5, 25, and 125 VPs/cell was determined at 48 h post-infection. Data are shown as mean ± SD for triplicate wells.

Since hexon constitutes the major part of adenovirus capsid, hexon modification may compromise viral growth characteristics and cause instability of the modified virion. Therefore, we performed thermostability assays and growth kinetic assays with our respective recombinant adenoviruses to test whether peptide incorporation into HVRs affected the structural integrity of the virions. Hexon-modified vectors, as well as control vectors, were subjected to heating at 45°C for different time intervals before infecting HEp-2 cells. Then the infectivity remaining at each time point was re-determined by fluorescent focus assay. All of the modified viruses showed similar stability to the unmodified viruses (Fig. 2A). The growth curves demonstrated that the hexon-modified viruses replicated with similar kinetics with unmodified Ad3EGFP (Fig. 2B). We also examined the efficiency of gene transfer for the hexon-modified viruses in HEp-2 cells with the aid of the EGFP reporter. There was no apparent difference between the modified viruses and the unmodified viruses, suggesting that the gene transfer ability was not significantly affected by hexon modification (Fig. 2C). These data suggested that the incorporation of SP70 epitope in HVR1, HVR2, or HVR7 of hexon did not significantly affect Ad fitness.

### Presentation of SP70 Epitope on Virion Surface

**Figure 3:**
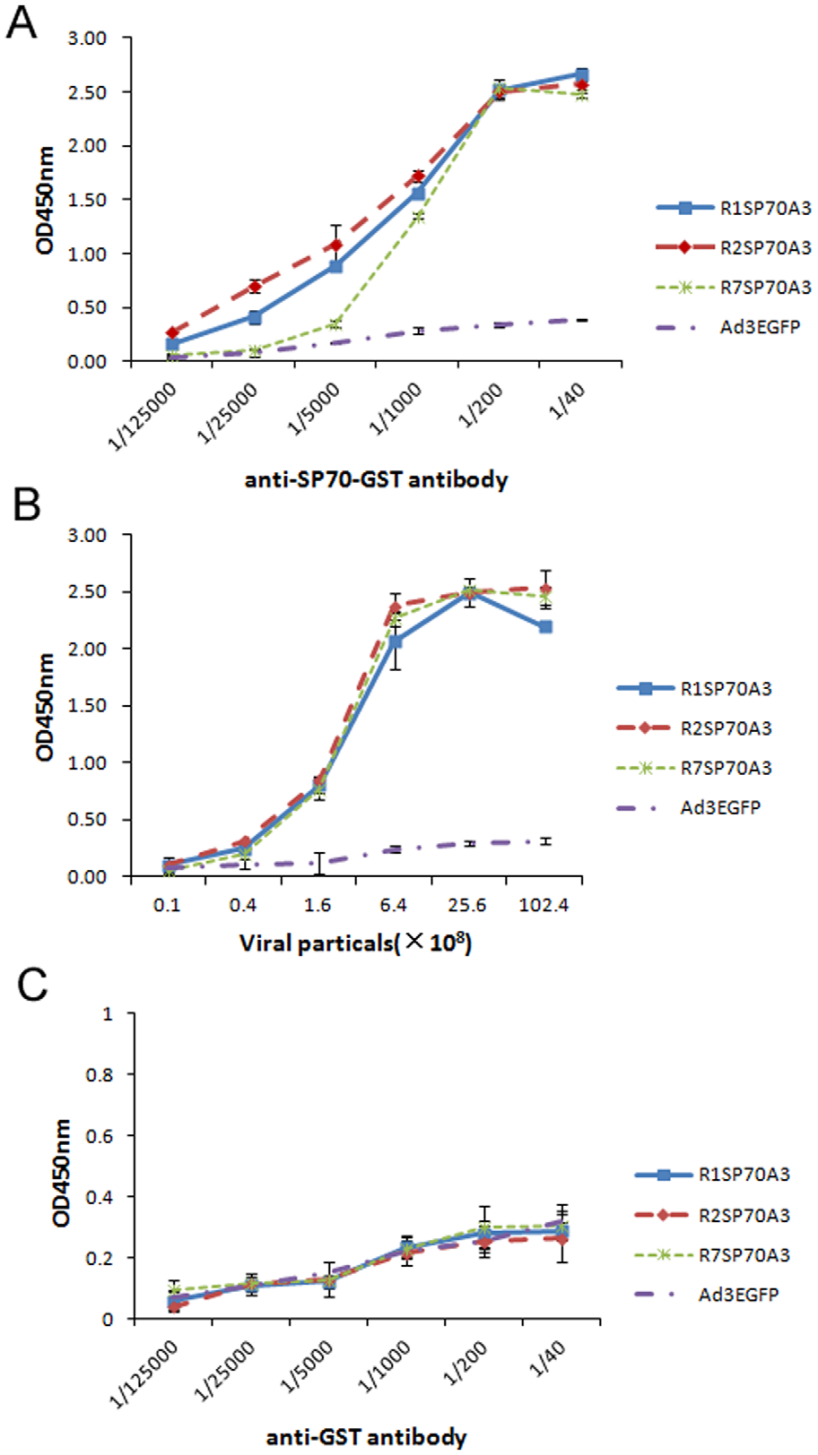
SP70 epitope incorporated in HVR1, HVR2, or HVR7 was exposed on the virion surface. (A) In the assay 5×10^8^ VPs/well of R1SP70A3, R2SP70A3, R7SP70A3, or Ad3EGFP were adsorbed on ELISA plates followed by varying dilutions of SP70-GST antiserum. (B) Various amounts of purified virions R1SP70A3, R2SP70A3, R7SP70A3 or Ad3EGFP were immobilized in the wells of ELISA plates and incubated with anti-SP70-GST antibody diluted at 1∶250. The binding was detected with an HRP-conjugated secondary antibody. (C) In the assay 5×10^8^ VPs/well of R1SP70A3, R2SP70A3, R7SP70A3, or Ad3EGFP were adsorbed on ELISA plates followed by varying dilutions of control GST antiserum. Data are shown as mean ± SD for triplicate wells.

To access whether the SP70 epitope was presented on the surface of modified virion, different amount of purified virions were immobilized on the wells of ELISA plates and incubated with anti-SP70-GST antibody. The results showed significant binding of the anti-SP70-GST antibody to the R1SP70A3, R2SP70A3, R7SP70A3, whereas no binding was seen in response to Ad3EGFP (Fig. 3A). These results indicated that the SP70 epitope was properly exposed on the virion surface when incorporated within HVR1, HVR2, or HVR7. In order to determine the capability of the SP70-specific antibody to bind capsid-incorporated antigen in a dose-dependent manner, a dose-response ELISA assay was performed with anti-SP70-GST antibody. A single concentration of R1SP70A3, R2SP70A3, or R7SP70A3 was applied to ELISA plates, followed by the addition of serial dilutions of anti-SP70-GST antibody. As predicted, the anti-SP70-GST antibody bound to all three modified virions in a dose dependent manner (Fig. 3B).

It was interesting to determine whether the anti-SP70-GST antibody could neutralize the modified rAds containing the SP70 epitopes. The SP70 peptide antiserum efficiently neutralized R1SP70A3, R2SP70A3, and R7SP70A3 with similar titers over 100-fold more than that against EV71 (Table 1).

**Table 1:** *In vitro* neutralization of hexon-modified adenoviruses.

Antiserum	Virus neutralization titer*				
	R1SP70A3	R2SP70A3	R7SP70A3	Ad3EGFP	EV71-08-02
Anti-SP70-GST	1440(±358)	1600(±0)	1440(±358)	<8	12.8(±4.4)
Anti-GST	<8	<8	<8	<8	<8
Anti-Ad3EGFP	4000	8000	8000	8000	<8

* Neutralization titers are expressed as the reciprocal of the lowest dilution of antiserum that blocks infection. Each experiment was repeated independently five times, and the mean values and SD are shown.

In order to test whether hexon modification could affect neutralization by anti-Ad3 antibody, HEp-2 cells were infected with hexon-modified rAds in the presence of Ad3EGFP-specific mouse antiserum. Ad infection was monitored by measuring EGFP expression. Replacement of HVR1 with the SP70 epitope clearly made the R1SP70A3 partly resistive to anti-Ad3EGFP serum, whereas the modification of HVR2 or HVR7 had no effect (Table 1).

### Anti-SP70 Antibodies Produced in Mice after Vaccination

**Figure 4:**
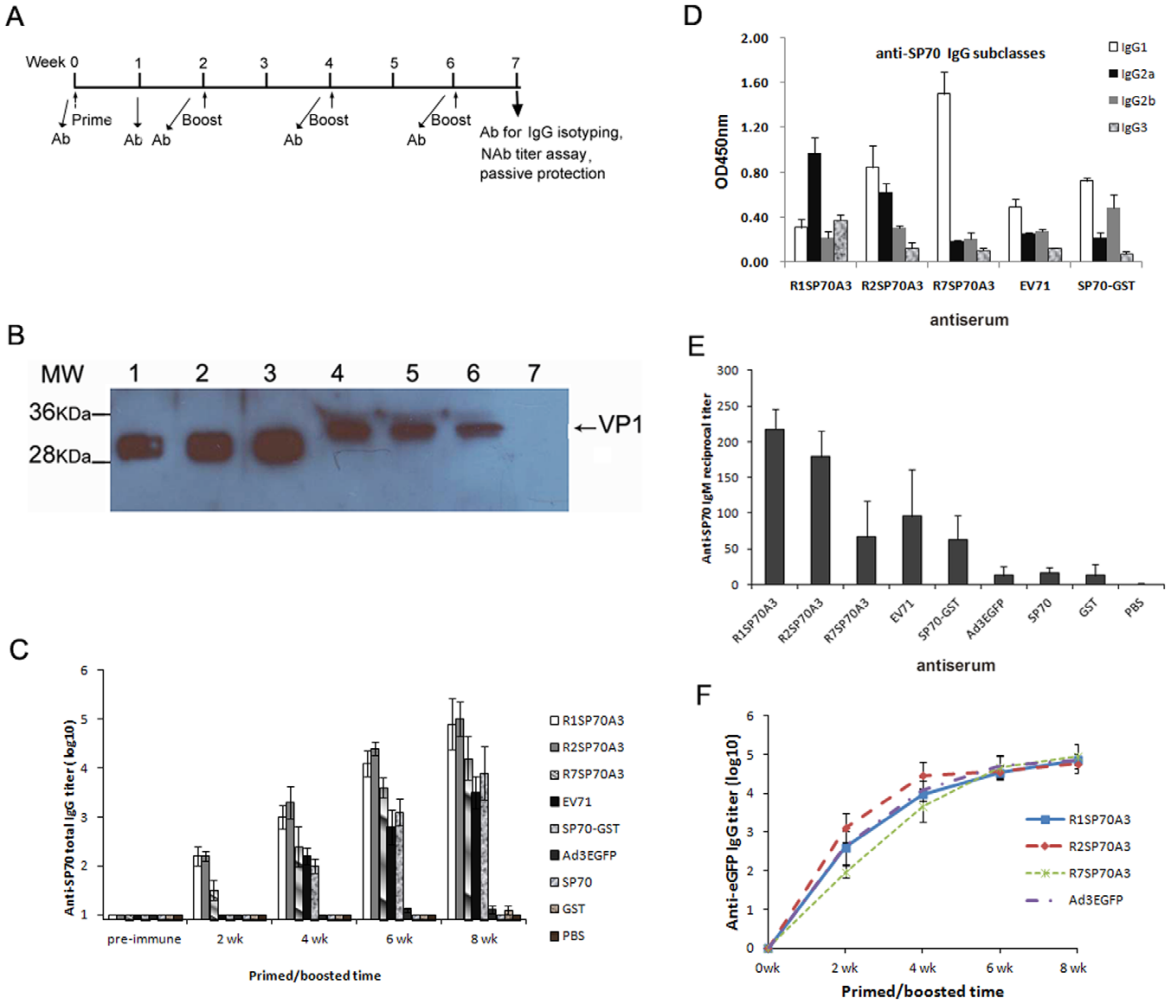
Adenoviruses expressing capsid-incorporated SP70 epitope elicited SP70-specific humoral immune responses. (A) Immunization regimen showing when immunizations were performed and when sera were collected for antibodies assay and passive protection experiment. (B) Western blot of purified SP70-GST protein (lane 1, 2, 3) and cell extracts from EV71-infected Vero cells (lane 4, 5, 6, 7). The proteins were transferred onto PVDF and reacted with mice antisera raised against R1SP70A3 (lane 1, 4), R2SP70A3 (lane 2, 5), R7SP70A3 (lane 3, 6), or Ad3EGFP (lane 7) respectively. Mice antibodies were detected with an HRP-linked secondary antibody and the signals were developed by ECL. The position of the EV71 VP1 capsid protein is indicated. (C) Total anti-SP70 IgG levels were quantified by indirect ELISA analysis with 1 µg/ml of synthetic SP70 peptide as antigen. Post-prime and post-boost sera were collected at 2, 4, 6, and 8 weeks (wk) for ELISA binding assays. Error bars represent the standard errors of the means per group (n = 5). (D) The distribution of SP70-specific IgG subtypes in the sera. Sera from mice boosted with the antigens were collected at 8-week for ELISA isotype binding assays. ELISA plates were coated with synthetic SP70 peptide. The plates were then incubated with immunized mice sera (anti-EV71-08-02 sera diluted 1∶1000 and other groups antisera diluted 1∶10000). The binding was detected with HRP-conjugated secondary isotype specific antibodies. OD at 450 nm represents isotype-specific SP70 antibody levels in sera. Data are shown as mean ± SD for each group mice (n = 4) for one of two independent experiments. (E) Early humoral responses to SP70 were determined by ELISA with synthesized SP70 peptide at day 7 following the first immunization with hexon-modified Ad vectors. Data are shown as mean ± SD for each group mice (n = 4). (F) Total anti-EGFP IgG levels were quantified by indirect ELISA analysis with 1 µg/ml of recombinant EGFP as antigen. Post-prime and post-boost sera were collected at 2, 4, 6, and 8 weeks (wk) for ELISA binding assays. Data are shown as means ± SD per group (n = 5).

To verify the immunizing potential of these vectors against EV71 we next sought to characterize the immune response to the peptide in mice. Equal numbers (5×10^9^ VPs) of the virions were used to immunize BALB/c mice. The sera were collected from mice at various days after priming and boosting (Fig. 4A). In order to evaluate the humoral responses qualitatively, Vero cells infected with EV71-08-02 strain and purified SP70-GST protein were subjected to Western blot analysis with antisera from mice immunized with R1SP70A3, R2SP70A3, R7SP70A3, or Ad3EGFP (Fig. 4B). Approximately 33 kDa protein bands associated with EV71 were detected with the antisera from mice immunized with modified virions. The size of the 32.6 kDa band corresponded to the expected size of the EV71 VP1 protein containing SP70 epitope. There was no VP1 protein detected with antiserum immunized with unmodified virion Ad3EGFP. These results indicated that humoral immune responses had been elicited against the SP70 incorporated in hexon.

Levels of anti-SP70 total IgG in sera of immunized mice were quantified using indirect ELISAs (Fig. 4C). Synthetic SP70 peptide was bound to ELISA plates. The plates were then incubated with the immunized mice sera. As expected, no IgG against SP70 was present in preimmune sera. In sera collected from control Ad3EGFP-injected mice or GST-injected mice at any time, no anti-SP70 IgG was detectable. Immunization with synthesized SP70 peptide or control PBS repeatedly also did not induce significant anti-SP70 IgG titers. On the contrary, mice immunized with rAds that contained capsid-incorporated SP70 epitope had detectable levels of SP70-specific IgG at 2 weeks, which increased approximately after boosting. Mice injected with heat-inactivated EV71 strain and recombinant protein SP70-GST had no detectable anti-SP70 IgG at 2 weeks, but had detectable levels of SP70-specific IgG at 4 weeks. Anti-SP70 IgG levels were significantly higher in rAds-injected mice than in EV71-injected mice (P<0.05). Serum levels of anti-SP70 total IgG were also quantified by ELISAs using plates coated with recombinant peptide SP70-GST, and the results were similar to that using synthetic SP70 peptide (data not shown). In summary, R1SP70A3, R2SP70A3, and R7SP70A3 elicited strong anti-SP70 humoral responses in vaccinated mice which increased after boosting.

Isotype-specific anti-SP70 IgG antibodies were produced in mice after vaccination. Levels of anti-SP70 IgG subtypes, IgG1 and IgG2a, IgG2b, IgG2c, IgG3 were also assessed in 8-week sera to determine the subtype of immune response being stimulated (Fig. 4D). The figure shows data of anti-EV71-08-02 sera diluted 1∶1000 and other groups antisera diluted 1∶10000. In mice, IgG1 is indicative of a Th2-type response, whereas IgG2a and IgG2b are predominantly produced during a Th1 response. As expected, mice receiving Ad3EGFP had no detectable levels of all anti-SP70 subtypes (data not shown). All mice had no detectable levels of anti-SP70 IgG2C (data not shown). Mice injected with heat inactivated EV71 or hexon-modified rAds had detectable levels of IgG1, IgG2a, IgG2b, and IgG3. Mice injected with SP70-GST had detectable levels of IgG1, IgG2a, and IgG2b without detectable levels of IgG3. These data suggested that both Th1- and Th2-type responses had been stimulated. However, R1SP70A3 immunization led to higher levels of IgG2a (OD≈0.97), but lower levels of IgG1 (OD≈0.31) and IgG2b (OD≈0.22). The R7SP70A3 and SP70-GST resulted in higher levels of IgG1 with lower levels of IgG2a and IgG2b. In contrast, the heat-inactivated EV71 and R2SP70A3 gave rise to relatively balanced levels of IgG1, IgG2a, and IgG2b.

The early IgM humoral responses against SP70 were assessed on day 7 after the first immunization. IgM titers increased two-fold in the sera of R1SP70A3 or R2SP70A3-immunized mice compared to those of mice immunized with R7SP70A3 (P<0.05), whereas for Ad3EGFP and PBS groups no SP70-specific IgM was detected (Fig. 4E).

Serum levels of anti-EGFP total IgG were also quantified using indirect ELISAs (Fig. 4F). After two injections, the transfer gene production EGFP-specific antibody responses were noticeably inhibited.

### Cellular Immune Response to Recombinant Adenovirus

**Figure 5:**
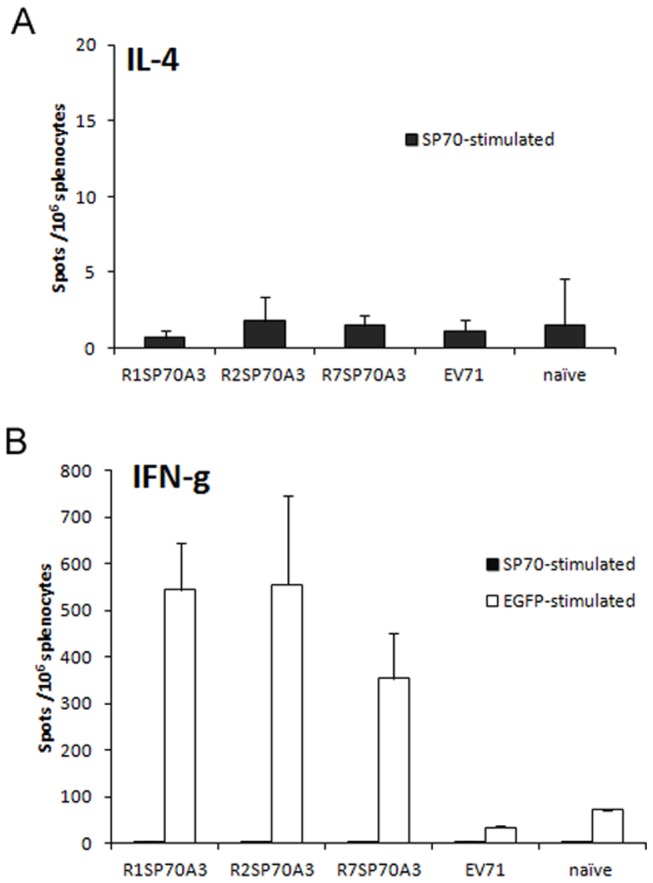
SP70-specific IL-4 and IFN-γ responses after immunized with the hexon-modified Ad3. Spleens were harvested from BALB/c mice (n = 2 each group) immunized with R1SP70A3, R2SP70A3, R7SP70A3, heat-inactivated EV71 or PBS on day 5 after the third injection. Lymphocytes were isolated from spleens by density gradient centrifugation and IL-4 and IFN-γresponses after in vitro stimulation with either SP70 or GFP protein were measured by ELISPOT analysis. Shown are data for the number of spot-forming cells per 1×10^6^ splenocytes after in vitro stimulation by subtracting the number of spots formed by unstimulated cells. Data represent the mean ± the SD of the mean of cells of each mouse plated in triplicate from one experiment representative of two separate experiments.

In this study we sought to determine if SP70 presented on the surface of adenovirus could be processed to activate specific cellular immunity. SP70-specific IL-4 and IFN-γ ELISPOT assays were performed on splenocytes of mice 5 days after the third injection (Fig. 5). In splenocytes obtained from heat-inactivated EV71 mice or rAds-injected mice, no IL-4- or IFN-γ-secreting cells were present above background levels after stimulation with SP70 peptide. In the case of rAds-injected mice, IFN-γ-secreting cells were detected upon stimulation with EGFP proteins (*P*<0.05). These results suggested that SP70-specific cellular responses were not significantly induced.

### Protection against Lethal EV71 Strain Challenge in Suckling Mice

**Figure 6:**
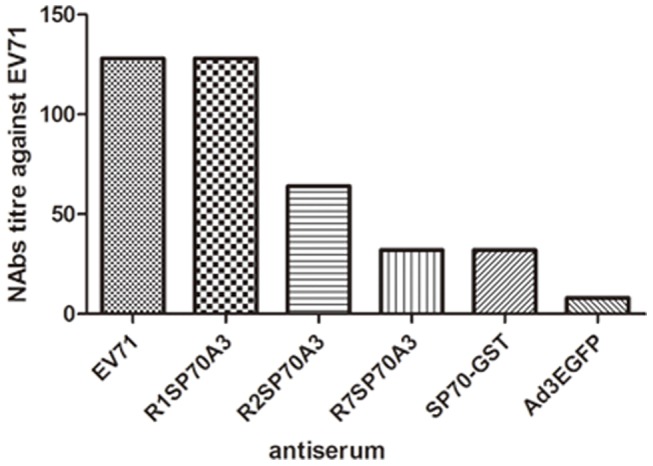
*In vitro* neutralization against EV71-08-02 strain. Two-fold serial dilutions of each antiserum collected at 8 week were incubated together with 1000 TCID_50_ of EV71-08-02 virus at 37°C for 1 h before added to Vero cells in 96-well plates. After incubation for 48h at 37°C, titers from triplicate wells were read as the highest dilution of serum that completely protected cells from CPE.

**Figure 7:**
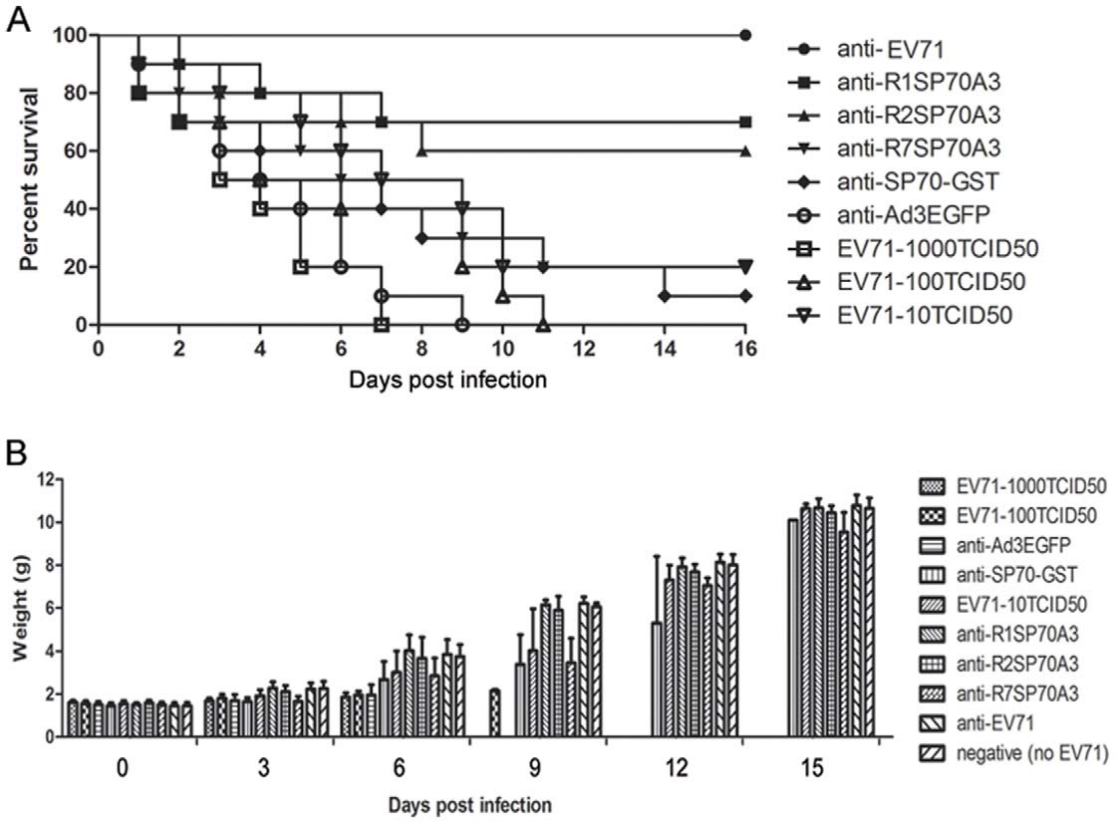
Passive protection against lethal EV71 strain challenge in suckling mice. (A) Groups of mice (n = 10 each group) at day 1 after birth were intraperitoneally injected with a lethal dose of 1000 TCID_50_ EV71 per mouse and 20 µl of heat-treated (56°C, 30 min) antiserum from EV71-, R1SP70A3-, R2SP70A3-, R7SP70A3- or Ad3EGFP-immunized mice. Suckling mice in the control group were not given any antiserum at all. Mice were monitored each day for body weight gain/loss and the occurrence of mortality until three weeks post-infection and mice that were failure in gaining weight or met end point criteria were humanely euthanized to minimize pain and suffering. Injection with EV71 (*P*<0.001), R1SP70A3 (*P* = 0.0012) and R2SP70A3 (*P* = 0.0042) antiserum significantly increased the survival rate compared with Ad3EGFP. (B) Monitoring of body weight gain/loss in suckling Balb/c mice after EV71 challenge. Error bars represent the standard errors of the means for all samples available of each group at that time point.

To assess the efficacy of passive protection by antisera raised against R1SP70A3, R2SP70A3, and R7SP70A3, suckling Balb/C mice born to naive dams were challenged with the EV71-08-02 strain (1000 TCID_50_ per mouse) administered with 20 µl of the respective antiserum. The titers of the respective antisera neutralizing EV71 were determined as the dilutions with complete protection from CPE in in vitro micro-neutralization assays (Fig. 6). The titers of the antisera obtained from heat-inactivated EV71, R1SP70A3-, R2SP70A3-, R7SP70A3- or SP70-GST-immunized mice were 128, 128, 64, 32 and 32 respectively. Antiserum from the Ad3EGFP-immunized mice did not confer any protection to Vero cells from CPE. Mice which were not infected did not show any sign of distress and all of them survived. Hence, these data demonstrated that the modified viruses R1SP70A3, R2SP70A3, and R7SP70A3 elicited anti-EV71 neutralizing antibodies. To determine the lethal viral dosage, groups of mice on day 1 after birth were infected intraperitoneally with different doses of EV71-08-02, ranging from 10 TCID_50_ to 1000 TCID_50_ per mouse. With an infective dose of 1000 TCID_50_ or 100 TCID_50_ virus, all mice died in two weeks post-infection, whereas a 10% survival rate was observed by day 21 post-infection for those mice infected with a reduced dosage of 10 TCID_50_ per mouse (Fig. 7A). 100%, 70%, 60%, 20%, and 10% survival rates (n = 10 each group) were observed by day 21 post-infection for groups of EV71-infected suckling mice that received the anti-EV71, anti-R1SP70A3, anti-R2SP70A3, anti-R7SP70A3 and anti-SP70-GST antiserum respectively, whereas EV71-infected suckling mice which did not receive any antiserum and those which received antisera from Ad3EGFP-immunized mice did not survive by day 16 post-infection (Fig. 7A). Compared with the group infected with EV71-1000TCID_50_ without antiserum, significant protective effects were observed for the groups that received anti-heat-inactivated EV71 (*P*<0.001), anti-R1SP70A3 (*P* = 0.0011), anti-R2SP70A3 (*P* = 0.0021) and anti-R7SP70A3 antiserum (*P* = 0.0297). Injection with anti-EV71 (*P*<0.001), anti-R1SP70A3 (*P* = 0.0012) and anti-R2SP70A3 (*P* = 0.0042) antiserum significantly increased the survival rate of EV71-infected suckling mice compared with Ad3EGFP antiserum. The body weights of mice that were protected against EV71 infectivity rose steadily up to an average of approximately 10.7 g at day 21 post-infection. At day 9 post-infection, they weighed 6.2 g on average when compared with unprotected mice that had an average body weight of 2.1 g before they died or were humanely euthanized (Fig. 7B).

**Figure 8:**
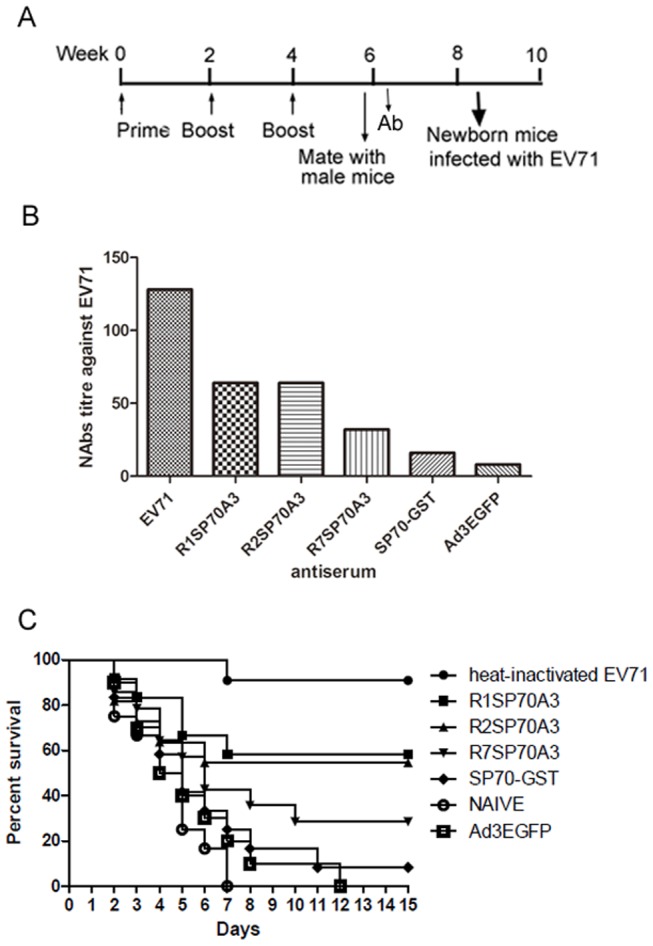
Protection to newborn mice against lethal EV71 challenge through maternally transferred antibodies. (A) Immunization timeline showing when immunizations were performed, sera were collected for antibodies assay and mice were allowed to mate with male mice. (B) *In vitro* neutralization against EV71-08-02 strain. Two-fold serial dilutions of each antiserum collected at 6 week from mother mice were incubated together with 1000 TCID_50_ of EV71-08-02 virus at 37°C for 1 h before added to Vero cells in 96-well plates. After incubation for 48 h at 37°C, titers from triplicate wells were read as the highest dilution of serum that completely protected cells from CPE. (C) 1-day-old neonatal BALB/c mice (n = 11 to 14) born to immunized dams were inoculated by i.p. with EV71 (1000 TCID_50_ per mouse) after fasting for 4 h. Mice were monitored each day for body weight gain/loss and the occurrence of mortality until three weeks post-infection and mice that were failure in gaining weight or met end point criteria were humanely euthanized to minimize pain and suffering. Effective protection were observed for the groups of which the mother mice were injected with R1SP70A3 (*P* = 0.0024), R2SP70A3 (*P* = 0.0178), R7SP70A3 (*P* = 0.0315) and heat-inactivated EV71 (*P*<0.001) compared with the group injected with PBS.

The vaccine candidates were also evaluated by challenging the neonatal mice born to immunized mother dams with lethal doses of EV71 (Fig. 8A). The NAbs titers of sera from the immunized mother mice at 6 week after the first injection were determined (Fig. 8B). No mice in the control groups receiving Ad3EGFP or only PBS survived by day 15 after challenge with a dose of 1000TCID_50_ EV71. In contrast, the survival rates by day 15 post-infection were 58.3% (7/12), 54.5% (6/11), 16.7% (4/14), 8.3% (1/12), and 90.9% (10/11) for the groups of which the mother dams were immunized with R1SP70A3, R2SP70A3, R7SP70A3, SP70-GST and heat-inactivated EV71, respectively (Fig. 8C). Compared with the group receiving only PBS, significant protective effects were observed for the groups that received R1SP70A3 (*P* = 0.0024), R2SP70A3 (*P* = 0.0178), R7SP70A3 (*P* = 0.0315) and heat-inactivated EV71 (*P*<0.001). The higher protection for the groups that received R1SP70A3 (*P* = 0.007), R2SP70A3 (*P* = 0.037) than Ad3EGFP demonstrated that R1SP70A3 or R2SP70A3 immunization conferred protection that was passed from the mother mice to the neonatal mice. These data indicated that the antibodies elicited by hexon-modified Ad3 conferred effective protection against lethal EV71 challenge.

## Discussion

**Figure 9:**
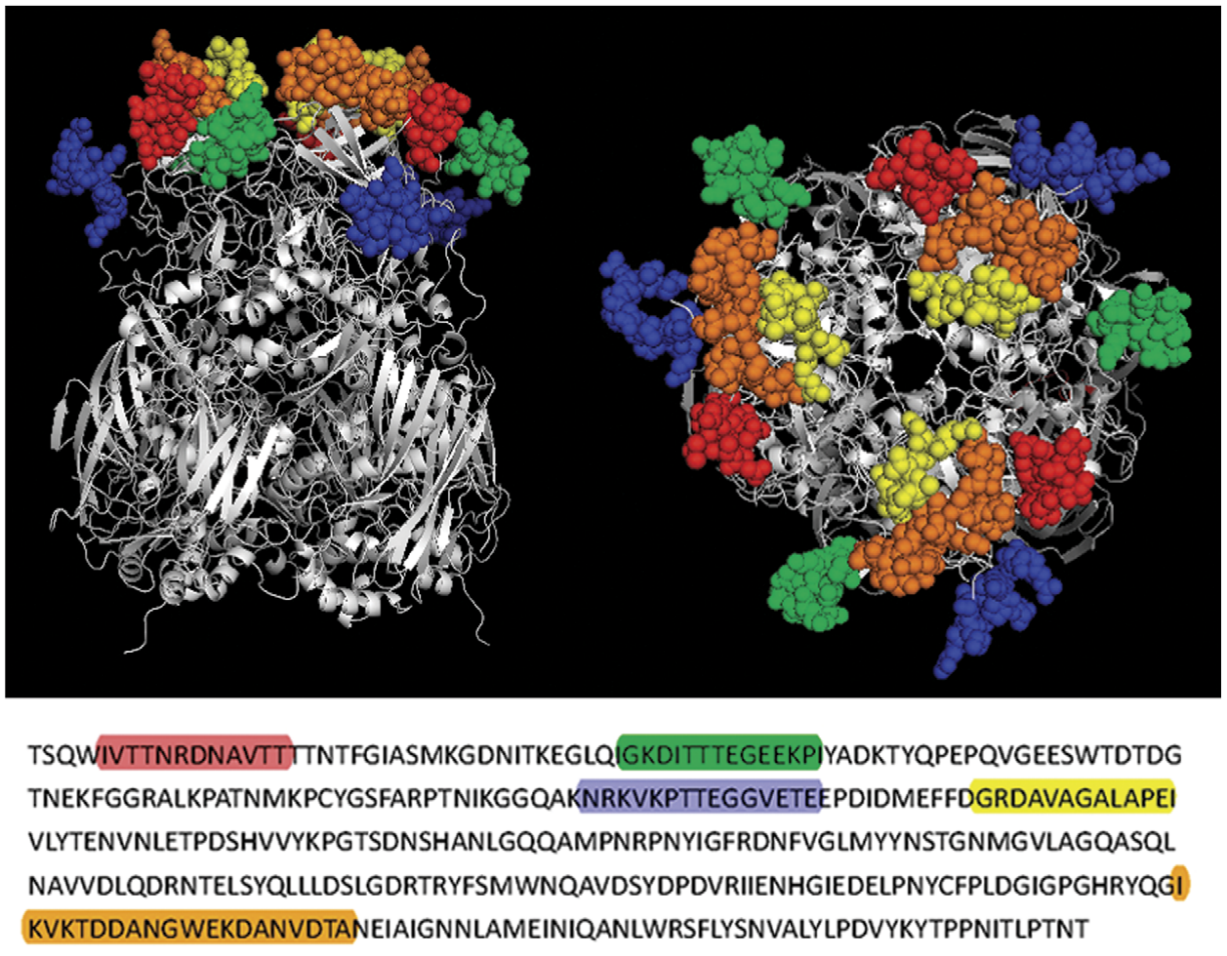
Molecular modeling of A3/H3 hexon and the five extruding domains predicted in the three towers region of hexon protein of A3/H3 colored by red, green, blue, orange and brown respectively (top view: left, side view: right) and their corresponding locations on the primary amino acid sequence of hexon.

The definition of HVRs used in the study is based on analysis performed by Crawford-Miksza and Rux et al. [33], [34]. By the web-FASTA tool of the PDB the chimpanzee adenovirus 68 (AdC68) hexon (PDB code: 2obe) at 2.1 Å resolution exhibited the highest degree of homology with the Ad3-gz01 hexon. Pichla-Gollon et al. have more precisely defined the five exposed surface loop regions based on the crystal structure of AdC68 hexon [35]. Yuan et al. also predicted five surface epitope polypeptide segments in HAd3-Harbin04B hexon by molecular modeling [36]. The five loops are located in HVR1, HVR2, HVR4, HVR5, and HVR7 respectively. In this study, we found that the SP70 peptide could be genetically inserted into HVR1, HVR2, and HVR7 regions of the Ad3 hexon without affecting virus formation and virion stability. However, the SP70 peptide inserted into HVR4 or HVR5 was affecting virus assembly or stability. The SP70 epitopes incorporated in HVR1, HVR2, and HVR7 appeared to be exposed on the virion surface as assessed by ELISA with purified whole virion (Fig. 3). These results indicate that HVR1, HVR2, and HVR7 of Ad3 hexon are suitable sites for antigen incorporation and display, since most peptide epitopes are between 6 and 14 amino acids in length. However, HVR2 and HVR5 of Ad5 vector are considered as the most flexible with respect to peptide or antigen incorporation [24], [37]. McConnell et al. also demonstrated that HVR5 of Ad5 can accommodate a peptide of up to 36 amino acids without adversely affecting virus infectivity, growth, or stability [23], [24]. The first possible reason may be that some amino acids in the replaced residue of HVR5 were indispensable to Ad3 hexon. Although the HVR4 loop is exposed on the surface of Ad3 hexon, it is located on the side, and may be involved in protein interactions in the intact virion (the molecular model of Ad3-gz01 hexon, Fig. 9). It is necessary to further investigate which amino acids of the HVR5 and HVR4 regions of Ad3 hexon could be deleted without affecting virion formation.

Anti-SP70 humoral responses could be evidently boosted after homologous Ad vector administration (Fig. 4C). In contrast, the transgene production EGFP-specific antibody responses were noticeably inhibited after two injections (Fig. 4F). These data indicate the advantage of the antigen capsid incorporation approach which is viable even in individuals with preexisting immunity to adenovirus. These are consistent with previous Ad5-based work [25], [38], [39]. Anti-vector immunity represents a key limitation of current Ad5 vectors based on gene transfer, because boosting against transfer gene production is ineffective if immunity against the Ad vector was present [29]. Shiratsuchi et al. [28] found that replacing Ad5 vector HVR1 with a malaria B cell epitope improved immunogenicity and circumvented preexisting immunity to adenovirus in mice. But we have an interesting finding that the peptide SP70 antiserum efficiently neutralized R1SP70A3, R2SP70A3, R7SP70A3 with a similar titer (Table 1). The data suggest that preexisting immunity to the foreign peptide incorporated in hexon should be taken into account which may inhibit transgene expression of gene transfer-based Ad vectors. It is a very interesting finding that the dominant SP70-specific IgG subtypes varied among the sera from mice immunized with rAds displaying SP70 in different HVRs (Fig. 4D). Continuous B cell stimulation by the antigen and maturation of high-affinity antibodies depend on the presence of T helper cells via the presentation of related peptides to the B cells [14]. This skewing toward Th1-type or Th2-type cellular immunity may account for the distribution of anti-SP70 IgG isotypes. In mice, IgG1 is indicative of a Th2-type response, whereas IgG2a and IgG2b are predominantly produced during a Th1 response. It suggests that the incorporation of foreign epitope into hexon may impact the cellular immune response to rAds, which may in part explain the difference of NAbs titers and protection efficacy against EV71 between R1SP70A3, R2SP70A3 and R7SP70A3. These may be a reflection of the Ad3 hexon T-cell epitopes or the adjuvant effect of the Ad vector. The finding that SP70-specific IL-4- or IFN-γ-secreting cells were not found in either rAds- or EV71 virions-immunized mice could be explained by two possibilities. First, it is possible that SP70 may not contain a T cell epitope. The previous study in which human CD4^+^ T-cell epitopes from VP1 of EV71 were identified did not find T-cell epitope in the region of SP70 [40]. Second, there may be a T cell epitope, but the responses elicited by it were too weak to be distinguished from background due to the sensitivity of the ELISPOT method used.

In recent years, various types of experimental vaccines against EV71 have been investigated, including heat-inactivated or formaldehyde-inactivated whole virion, live attenuated virions, virus-like particles, recombinant VP1 protein, DNA vaccine and peptide-based vaccine targeting the neutralizing domain [41], [42], [43], [44], [45], [46]. Four strong cross-reactivity inducing peptides between EV71 and human brain tissue were identified and two of them locate at VP2 and VP1, which may be responsible for the pathogenesis post infection [42], [47]. Several linear T-cell and B-cell epitopes of EV71 have been identified by different groups [9], [48]. Since EV71 infection caused no apparent clinical symptoms in adult mice, the neonatal mice model of EV71 infection has been used here and by several other groups to evaluate candidate vaccines [42], [45]. It will be helpful for vaccine evaluation to develop a trangenic adult mice model of EV71 infection or establish a simian model in which the clinical and neuropathological symptoms are similar to human cases of EV71 infection [49]. Three main types of EV71 can be further divided into 11 subtypes: A, B1–B5 and C1–C5 [50], [51]. C type EV71 was responsible for most of the infection cases in recent years in Asia, and C4 subtype EV71 was the main strain in mainland China [5], [52]. Consistent with previous studies, EV71-neutralizing antibodies elicited by inactivated EV71 particles offered the most effective protection against EV71 [10], [44], [53]. Strikingly, the anti-SP70 IgG titers were over 10-fold higher following immunization with hexon-modified Ad3 vectors compared with immunization with recombinant protein SP70-GST or heat-inactivation EV71 (Fig. 4C). These data illustrate the advantage of the potent immunologic properties of the adenovirus hexon for presenting peptide epitopes to the immune system. The antisera raised against R1SP70A3 have the highest NAbs titers against EV71 compared with the other hexon-modified Ad3 vectors (Fig. 6). The anti-Ad total IgG titers were comparable for all rAds (data not shown). It is likely that the SP70 epitope inserted into HVR1 of Ad3 hexon may be presenting in the more similar conformation to that within the context of an EV71 virion. The nature of epitopes and the precise sites of insertion could account for the observed difference [19], [25].

In conclusion, we have successfully incorporated a 15 amino acid linear neutralizing epitope SP70 derived from EV71 into the HVR1, HVR2, and HVR7 of Ad3 hexon and presented it on the surface of virions. The SP70s presenting on the Ad3 surface elicited neutralizing antibodies which were able to confer good *in vivo* protection against EV71 in suckling Balb/c mice. Together, these results indicate that the adenovirus type 3 is an effective vehicle for delivering and presenting peptides to the immune system. This approach is useful for the design of epitope-based vaccines for pathogens and cancers. In the next step, we will set out to create novel multivalent Ad3 vaccine vectors presenting several linear T-cell or B-cell epitopes derived from EV71 on the same virion that would yield optimal vaccine efficacy.

## Materials and Methods

### Cells, Virus Strains, and Vectors

A sub-cell line of AD293 and HEp-2 cells (human Epidermoid carcinoma cell line), Vero cells (African green monkey kidney cell lines) purchased from ATCC were kept in our lab and cultured in DMEM supplemented with 10% FBS and antibiotics [29], [54]. The E3-defective adenovirus type 3 replication-competent plasmid, pBRAdV3dE3egfp (pAd3egf) with enhanced green fluorescence protein (EGFP) as report gene, corresponding virus Ad3/H3 (Ad3EGFP) and the shuttle vector pBRH3S were provided by Qiwei Zhang or constructed before [29], [54]. The EV71-08-02 strain (GenBank No. FJ360545), a clinical isolate of EV71 virus from a child with HFMD in 2008 in Guangzhou of southern China was obtained from Guangzhou Woman and Children’s Medical Center and determined to be genotype C4 by genome sequence analysis. EV71 virus stock was collected from infected Vero cells 3 days post-infection at 37°C. The virus from the cell culture was purified by precipitation with 7% polyethylene glycol 8000 and then centrifuged on a 30% sucrose cushion at 25,000×g for 4 h. The virus titer was determined as TCID_50_ in Vero cells, based on typical cytopathic effect (CPE) produced by viral infection.

### Construction of Recombinant Adenoviral Vectors

**Figure 10:**
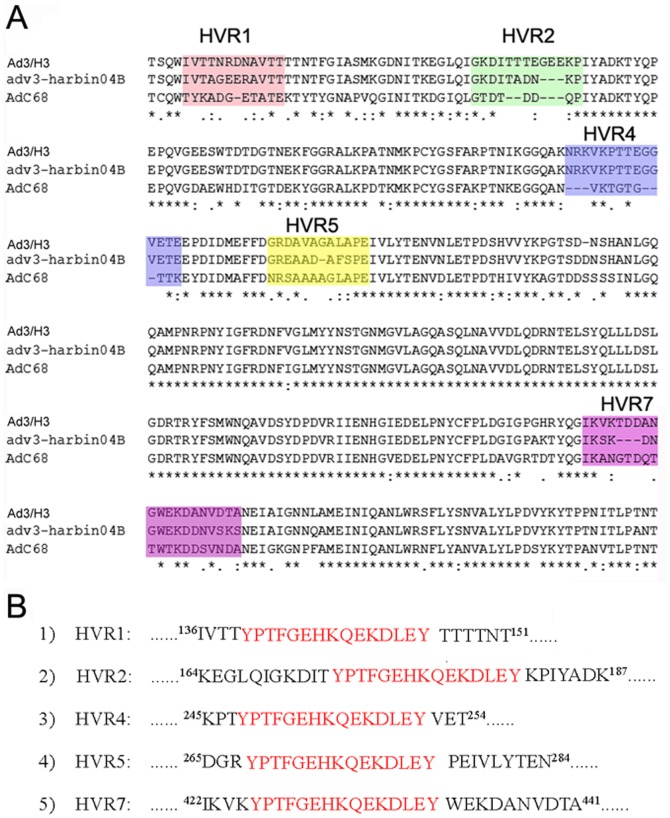
Diagram of the SP70 incorporation sites in Ad3 hexon. (A) Partial amino acid sequence alignments of Ad3-gz01 and AdC68, Ad3-Harbin04B hexons, showing residues 133–490 of Ad3-gz01 hexon. The potential surface epitope polypeptide segments are colored. (B) Amino acid residues of the SP70 motif marked in red were incorporated into HVRs of Ad3 hexon. The numbers show the positions of the amino acid residues in the Ad3 hexon.

According to the alignment with amino acid sequences of AdC68 and Ad3-Harbin04B strain hexons, it is highly likely that HVR1, HVR2, HVR4, HVR5, and HVR7 of Ad3/H3 hexon extrude from the capsid surface and stretch to the external environment (Fig. 10A). The amino acid residues of SP70 containing 208–222 of VP1 protein are highly conserved amongst the VP1 sequences of EV71 strains from various subgenogroups [9], [10]. The EV71 neutralizing epitope SP70 was molecularly incorporated into these hexon HVRs at positions marked in Fig. 10B.

To accomplish these genetic modifications, we first obtained hexon fragments containing sequences encoding the SP70 epitope in different HVRs via overlapping PCR. For instance, to obtain a SP70 insertion in HVR1 (R1SP70), using Ad3 hexon as the template, we first amplified fragment 1L with primers HexU and hr1-sp70R, and fragment 1R with primers HexD and hr1-sp70U (shown in Table 2). After purification of fragments 1L and 1R, 50 ng of each fragment was mixed and used as the template and primers for 6 cycles of amplification, resulting in insertion of sequences encoding SP70 and the spacers in HVR1. Next, primers HexU and HexD were added into the tubes to amplify the R1SP70 fragment. Other insertions were obtained in the same way with corresponding primers (Table 2).

**Table 2:** Table **2.** Primers used to incorporate the SP70 epitope into the HVRs of the Ad3 hexon.

Name	Sequence*
HexU	5′-CCCATCGATGATGCCCCAATGGGCA-3′
HexD	5′-CCGGGATCCACCTCAAAAGTCATGTCCAGC-3′
hr1-sp70U	5′-GTTACAACGTATCCCACATTCGGAGAACACAAACAGGAGAAAGATCTTGAATATACTACCACCACAAACACATTTGGC-3′
hr1-sp70R	5′-GGTGGTAGTATATTCAAGATCTTTCTCCTGTTTGTGTTCTCCGAATGTGGGATACGTTGTAACTATCCACTGAGATG-3′
hr2-sp70U	5′-GACATTACCTATCCCACATTCGGAGAACACAAACAGGAGAAAGATCTTGAATATAAGCCCATTTATGCCGATAAAAC-3′
hr2-sp70R	5′-AATGGGCTTATATTCAAGATCTTTCTCCTGTTTGTGTTCTCCGAATGTGGGATAGGTAATGTCTTTCCCAATTTGCA-3′
hr4-sp70U	5′-AAACCAACATATCCCACATTCGGAGAACACAAACAGGAGAAAGATCTTGAATATGTTGAAACTGAGGAACCAGATA-3′
hr4-sp70R	5′-TTCAACATATTCAAGATCTTTCTCCTGTTTGTGTTCTCCGAATGTGGGATATGTTGGTTTTACTTTTCTGTTTTTAGC-3′
hr5-sp70U	5′-GATGGTAGGTATCCCACATTCGGAGAACACAAACAGGAGAAAGATCTTGAATATCCTGAAATTGTGCTTTATACGGA-3′
hr5-sp70R	5′-AATTTCAGGATATTCAAGATCTTTCTCCTGTTTGTGTTCTCCGAATGTGGGATACCTACCATCGAAAAATTCCATATC-3′
hr7-sp70U	5′-AAAGTTAAATATCCCACATTCGGAGAACACAAACAGGAGAAAGATCTTGAATATTGGGAAAAAGATGCTAATGTTGAT-3′
hr7-sp70R	5′-TTTCCCAATATTCAAGATCTTTCTCCTGTTTGTGTTCTCCGAATGTGGGATATTTAACTTTAATGCCTTGATACCT-3′

* Underlined letters represent the sequences encoding the SP70 epitope.

The hexon fragments containing SP70 epitope were purified and subcloned into Ad3 hexon shuttle vector pBRH3S with ClaI and BamHI restriction enzymes. The resultant shuttle plasmids were named R1SP70-pBRH3S, R2SP70-pBRH3S, R4SP70-pBRH3S, R5SP70-pBRH3S, R7SP70-pBRH3S, respectively. To create Ad3 vectors containing SP70 epitope in the HVRs of hexon, these shuttle plasmids were digested with EcoRI and SalI, and the fragments containing the homologous recombination regions and the hexon genes were purified, and then recombined with AvRII- and PacI- double-digested pAd3egf in *Escherichia coli* strain BJ5183. The resultant clones containing SP70 and EGFP gene were designated pR1SP70A3egf, pR2SP70A3egf, pR4SP70A3egf, pR5SP70A3egf, pR7SP70A3egf and selected by PCR using primers HexU and SP70r (5′-GAT CTT TCT CCT GTT TGT GTT CTC C-3′), egfu (5′-GAA ATC GAT GTG AGC AAG GGC GAG GAG CT-3′) and egfr (5′-CTA GAT CCG GTG GAT CCC-3′). The constructs were confirmed by restriction digestions and sequencing analysis.

To rescue viruses, these modified plasmids were digested with AsisI to linearize genomic DNA and then transfected into AD-293 cells grown in 30 mm dishes using Lipofectamine LTX with Plus reagents (Invitrogen, USA). The transfected cells were cultured at 37°C/5% CO_2_ for 6–10 days and were examined daily for evidence of CPE. Cells were frozen and thawed for three cycles and fresh cultures of HEp-2 cells were infected with viral suspension. At 96 hours post-infection, the virus was harvested and designated R1SP70A3, R2SP70A3, R4SP70A3, R5SP70A3, R7SP70A3. Finally, a total of twenty 100 mm dishes containing HEp-2 cells infected with modified Ad3 were harvested, followed by purification by standard CsCl gradient centrifugation, as described by Wu [27]. The virus particle (VPs) titers were determined by spectrophotometry using a conversion factor of 1.1×10^12^ VPs per absorbance unit at 260 nm. Purified DNA from CsCl preparations of viruses were used for PCR with primers HexU and SP70r, Hu1 (5′-CAR TGG KCR TAC ATG CAC ATC-3′) and Hr1 (5′-CCM GCR TTR CGG TGG TGG TT-3′). The full length modified hexon genes of the viruses were identified by sequencing.

### Growth Characteristics and Virus Thermostability

Growth curves were generated by infecting HEp-2 cells in 24-well plates with Ads at 5 VPs/cell in 500 µl growth medium containing 2% FBS. The infected cells were harvested with medium at various time points post-infection for 4 days. The collected cells together with the medium were lysed by three freeze–thaw cycles, and subjected to centrifugation at 10000 g for 30 min at 4°C for cell debris removal. The number of infectious particles in each sample was determined by fluorescent focus assay at 48 h post-infection, and plotted as growth curves. To test the heat stability of the hexon-modified adenovirus, the viruses were incubated at 45°C for 0, 5, 10, 20, 40 or 60 min in growth medium containing 2% FBS before infecting HEp-2 cells. Then the infectivity remaining at each time point was re-determined by fluorescent focus assay at 48 h post-infection.

For gene transfer efficacy assay, HEp-2 cells were seeded into 96-well NUNC Optical bottom plates (ThermoFisher Scientific) and infected with Ads at multiplicities of infection (MOI) of 1, 5, 25, and 125 VPs/cell, and cultured in RPMI Medium 1640 (GIBCO) without phenol red and without serum for 48 h. The fluorescence was measured with a Varioskan Flash Multimode Reader (Thermo Scientific) by exciting at 488 nm and recording the emitted light at 507 nm. Background fluorescence was equalized to wells only containing cells.

### Peptide Synthesis and Production of Recombinant SP70 Fusion Peptide

The peptide sp70 was synthesized by Jetway Co. Ltd (Guangzhou, China). The peptide was purified by high-performance liquid chromatography to 85% purity, and the identity of the peptide was confirmed by mass spectrometry analysis. SP70 was dissolved in water to 1 mg/ml and stored at −80°C in aliquots to avoid repetitive freeze-thaw cycles.

To produce a recombinant SP70 fusion peptide with an N-terminal GST tag, the 126 bp of the fragment containing SP70 gene was amplified with primers E592u (5′-CGT GAA TTC GCG AGT GCT TAT CAA TGG-3′) and E696r (5′-CGA TGC GGC CGC CGT GCC CAT CAT GTT GTT-3′) by RT-PCR using EV71 genome as template. Then the fragment was cloned into the expression vector pGEX-4T-3 with an N-terminal GST tag after EcoRI- and NotI- double-digestion. Then the recombinant vector pGEX-SP70 was transformed into *Escherichia coli* BL21(DE3). The recombinant fusion peptide SP70-GST was purified by affinity chromatography using GST-Bind Resin (Novagen, USA) from a single transformant under native conditions. The control protein GST was purified at the same condition.

### Mouse Immunization

Female Balb/c mice aged 4–6 weeks were used in the immunization experiments. Mice in groups of 5 were immunized with 5×10^9^ VPs recombinant viruses Ad3EGFP, R1SP70A3, R2SP70A3, or R7SP70A3 by intramuscular (i.m.) injection, heat-inactivated EV71 strain (10 µg total protein), 50 µg SP70-GST or GST protein and synthesized SP70 peptide with complete adjuvant by intraperitoneal (i.p.) inoculation, and phosphate-buffered saline (PBS) served as the negative controls. Three booster doses were given at 2 weeks intervals with the same dose of antigen. Immune sera were collected from anesthetized mice before each immunization and kept frozen for serology tests. Blood was collected from anesthetized mice via the orbit at ten days after the final immunization and transferred to a microfuge tube. Tubes were centrifuged and sera were removed and frozen at −80±5°C for in vitro neutralization assay and passive protection experiment against lethal EV71 strain challenge in suckling mice.

### Western Blot Analysis

To analyze SP70 presentation on selected vectors, the total proteins of purified EV71 or recombinant adenoviruses cultures were separated in 10% SDS polyacrylamide gels. The proteins were transferred onto PVDF membranes using a wet blotter. The membranes were blocked with 5% skim-milk in PBS and then incubated with the anti-SP70-GST serum (1∶250), washed and exposed to a 1∶10000 dilution of goat anti-mouse IgG (H + L)-HRP conjugate affinity-purified secondary antibody (Bio-Rad). The blot was developed using Immobilon Western chemiluminescence HRP substrate (Millipore).

Total viral proteins of the EV71 strain and purified SP70-GST were used to study immunoreactivity of the antisera of mice immunized with R1SP70A3, R2SP70A3, and R7SP70A3. Briefly, proteins were separated in 12% SDS polyacrylamide gels, and transferred onto PVDF membranes. After blocking with 5% skim-milk, the membranes were incubated with the hyperimmune anti-rAds sera, washed and exposed to a 1∶10000 dilution of secondary Ab (Bio-Rad). The blot was developed using the substrate described above.

### ELISA

In order to determine if the SP70 peptide was surface exposed on the rAds virion, whole virus ELISA was performed according to the method previously described by Matthews et al. (30). Briefly, 96-well plates (Nunc Maxisorp) coated overnight with different amounts of viruses ranging from 10^7^ to 10^10^ VPs in 100 µl of 100 mM carbonate buffer (pH 9.6) per well at 4°C were washed with 0.05% Tween 20 in Phosphate-buffered saline (PBST) and blocked with 2% bovine serum albumin (BSA) in PBST. Then the plates were incubated with anti-SP70-GST antibody for 1 h at 37°C. The plates were washed three times with PBST and incubated for 1 h with a 1∶8000 dilution of secondary Ab. The plates were washed four times, developed with tetramethylbenzidine (TMB) substrate, stopped with 2 M H_2_SO_4_, and analyzed at 450 nm with ELISA plate reader (Thermo Scientific Multiskan MK3).

For the anti-SP70 humoral responses, ELISA plates were coated with 0.1 µg of the synthetic SP70 peptide or recombinant SP70-GST protein in 100 µl of 50 mM carbonate buffer (pH 9.6) per well. Plates were washed and then blocked with 2% BSA/PBST. After incubation with serially diluted sera for 1 h at 37°C, the plates were washed three times with PBST and incubated for 1 h with a 1∶8000 dilution of secondary Ab. Then the plates were developed with TMB. In order to determine SP70 isotype-specific reactivity of the mice anti-sera, ELISA plates were coated with 0.1 µg of the synthetic SP70 peptide in 100 µl of 50 mM carbonate buffer (pH 9.6) per well and then blocked with 2% BSA/PBST. After incubation with serially diluted sera for 1 h at 37°C, the plates were washed three times with PBST and incubated for 1 h with a 1∶2000 dilution of peroxidase-conjugated Affinipure goat anti-mouse sub-types specific antibody (ProteinTech). ELISAs were developed with TMB substrate and measured at 450 nm with ELISA plate reader. Each assay was performed three times independently. Endpoint titer was defined as the highest dilution at which the OD_450_ was at least 0.1 above that of wells receiving no serum. Wells receiving no serum always had an OD_450_ of <0.1.

### *In vitro* Neutralization

The presence of neutralizing antibodies against EV71 was assayed in an *in vitro* microneutralization test with Vero cells. Aliquots of mice sera were incubated at 56°C for 30 min prior to use. Briefly, 50 µl of two-fold serially diluted mice sera were mixed with equal volumes of 1000 TCID_50_ of virus and incubated at 37°C for 1 h. Then the mixtures were adsorbed onto 96-well microtiter plates seeded the previous day with Vero cells. After incubation for 48 h at 37°C, titers from triplicate wells were read as the highest dilution of sera that inhibited virus growth.

For the *in vitro* Ad neutralization experiments, the antisera from mice immunized with Ad3EGFP were 2-fold serially diluted in DMEM, and 50 µl aliquots of each dilution were mixed with 50 µl rAds of 2×10^5^ VPs. The antibody-virus mixtures were incubated for 1 h at 37°C and then transferred to 96-well plates containing 85–95% confluent monolayers of HEp-2 cells. Monolayers were cultured in RPMI Medium 1640 (GIBCO) without phenol red and serum for 72 h. Infected cells were analyzed with a Varioskan Flash Multimode Reader (Thermo Scientific) to measure the EGFP expression. The serum neutralization titer was defined as the reciprocal of the serum dilution that inhibited 90% of the EGFP expression.

### ELISPOT

For assessment of the SP70-specific cellular immune response, Babl/c mice were immunized intramuscularly with 5×10^9^ VPs recombinant viruses R1SP70A3, R2SP70A3, or R7SP70A3. Two booster doses were given at 2 weeks intervals. Heat-inactivated EV71 strain (10 µg total protein) and PBS (naive) with adjuvant were injected into mice by i.p. served as the controls. The frequency of antigen-specific T lymphocytes was determined by IFN-γ and IL-4 ELISPOT assays 5 days after the third injection. The spleens of two mice from each group were collected and homogenized in a density gradient of 1.081 g/mL EZ-Sep™ medium (DAKEWE Biotech), then the cell suspensions were transferred into 15 ml centrifugal tubes and covered with 200 µl RPMI-1640 medium, centrifuged at 800×g for 30 min at room temperature. The mononuclear-cell-enriched fraction was collected, then washed once and diluted to the desired concentration with RPMI-1640 medium. 5×10^5^ cells per well were cultured in Lympho-Spot™ serum free medium universal (DAKEWE Biotech) and stimulated with 20 µg/ml SP70 peptide or 10 µg/ml GFP protein, or left unstimulated as control for 24 hr in 96-well plate that had been coated with capture antibodies to either mouse IFN-γ or IL-4. After 24 hr, the splenocytes were removed, and IFN-γ or IL-4 secretion was detected as recommended by the manufacturer (DAKEWE Biotech). The spots were counted using an ELISPOT reader. The number of spot-forming cells for each stimulated sample was determined by subtracting the number of spots formed by unstimulated cells from the same mice. All samples were run in triplicate.

### *In vivo* Protection against Lethal Enterovirus 71 Infection

EV71 infection caused no apparent clinical symptoms in adult Balb/c. For the passive protection study, neonatal Balb/c mice were used as described by Foo et al. [10]. The sera from mice on day 10 after the 4th immunization were collected as described above (Fig. 4A). Groups of neonatal mice (n = 10 each group) at day 1 after birth were injected intraperitoneally with 10 µl of EV71 (1000 TCID_50_ per mouse) and with 20 µl of heat-treated (56°C, 30 min) mice immune sera one hour later. Suckling mice (n = 10 each group) from control groups were either given anti-Ad3EGFP serum or not given any antiserum at all. Mice were monitored each day for body weight gain/loss and the occurrence of mortality until three weeks post-infection and mice that were failure in gaining weight or met end point criteria were humanely euthanized to minimize pain and suffering.

Protective effect of vaccine candidates against EV71 infection was also evaluated in newborn Balb/c mice with the transferred maternal antibody from immunized mother mice. Female Balb/c mice aged 6 weeks were immunized with 1×10^10^ VPs recombinant viruses Ad3EGFP, R1SP70A3, R2SP70A3, or R7SP70A3 by i.m. injection, heat-inactivated EV71 strain (10 µg total protein), 50 µg SP70-GST protein with adjuvant by i.p. inoculation, and phosphate-buffered saline (PBS). Animal were boosted twice after 2 weeks and 4 weeks of the first immunization. The sera were collected from the mother mice 2 week after the third injection and then sent to in vitro NAbs titer analysis (Fig. 8A). The female mice were allowed to mate at 5 weeks after the first injection (Fig. 8A). For EV71 challenge, groups of 1-day-old pups born to the immunized dams were inoculated by i.p. with EV71 (1000 TCID_50_ per mouse) after fasting for 4 h. Mice were monitored each day for body weight gain/loss and the occurrence of mortality until three weeks post-infection and mice that were failure in gaining weight or met end point criteria were humanely euthanized to minimize pain and suffering.

### Statistical Analysis

The data are presented as the mean ± the standard error. Statistical significance was determined using Prism 5.0 software. Comparisons among groups were performed by ANOVA with Bonferroni’s test to account for multiple comparisons. Differences in Kaplan-Meier survival curves between the groups were assessed using the log-rank test. P values of less than 0.05 were considered as statistically significant.

### Ethics Statement

Samples with written informed consent in this study were taken as part of standard care. The experiments involving patient and all animal procedures in this study were reviewed and approved by the First Affiliated Hospital of Guangzhou Medical University Ethics Committee. The animal experiments were conducted in strict accordance with the recommendations in the Guide for the Care and Use of Laboratory Animals of the National Institutes of Health. To minimize suffering after viral challenge, mice were monitored each day and mice that were failure in gaining weight or met end point criteria were humanely euthanized by administration of pentobarbital sodium.
