# Correction: Protection against Enterovirus 71 with Neutralizing Epitope Incorporation within Adenovirus Type 3 Hexon

**DOI:** 10.1371/annotation/2da932d4-8654-45c8-bf83-7b3b09dbc146

**Published:** 2012-10-11

**Authors:** Xingui Tian, Xiaobo Su, Xiao Li, Haitao Li, Ting Li, Zhichao Zhou, Tianhua Zhong, Rong Zhou

There is an error in affiliation #1 for authors Xingui Tian, Xiao Li, Haitao Li, Ting Li, Zhichao Zhou, Tianhua Zhong, and Rong Zhou. The correct affiliation #1 is:

State Key Laboratory of Respiratory Diseases, The First Affiliated Hospital of Guangzhou Medical College, Guangzhou Medical College, Guangzhou, Guangdong, China. 

